# Long Pentraxin 3-Mediated Fibroblast Growth Factor Trapping Impairs Fibrosarcoma Growth

**DOI:** 10.3389/fonc.2018.00472

**Published:** 2018-11-01

**Authors:** Priscila Fabiana Rodrigues, Sara Matarazzo, Federica Maccarinelli, Eleonora Foglio, Arianna Giacomini, João Paulo Silva Nunes, Marco Presta, Adriana Abalen Martins Dias, Roberto Ronca

**Affiliations:** ^1^Laboratory of Inflammation and Cancer, Department of General Biology – ICB, Universidade Federal de Minas Gerais, Belo Horizonte, Brazil; ^2^Department of Molecular and Translational Medicine, School of Medicine, University of Brescia, Brescia, Italy

**Keywords:** long pentraxin-3, FGF-trap, fibrosarcoma, FGF, FGFR

## Abstract

Fibrosarcomas are soft tissue mesenchymal tumors originating from transformed fibroblasts. Fibroblast growth factor-2 (FGF2) and its tyrosine-kinase receptors (FGFRs) play pivotal roles in fibrosarcoma onset and progression, FGF2 being actively produced by fibroblasts in all stages along their malignant transformation to the fibrosarcoma stage. The soluble pattern recognition receptor long pentraxin-3 (PTX3) is an extrinsic oncosuppressor whose expression is reduced in different tumor types, including soft tissue sarcomas, via hypermethylation of its gene promoter. PTX3 interacts with FGF2 and other FGF family members, thus acting as a multi-FGF antagonist able to inhibit FGF-dependent neovascularization and tumor growth. Here, PTX3 overexpression significantly reduced the proliferative and tumorigenic potential of fibrosarcoma cells *in vitro* and *in vivo*. In addition, systemic delivery of human PTX3 driven by the Tie2 promoter inhibited the growth of fibrosarcoma grafts in transgenic mice. In a translational perspective, the PTX3-derived small molecule FGF trap NSC12 prevented activation of the FGF/FGFR system in fibrosarcoma cells and reduced their tumorigenic activity *in vivo*. In conclusion, impairment of the FGF/FGFR system by FGF trap molecules may represent a novel therapeutic approach for the treatment of fibrosarcoma.

## Introduction

Soft tissue sarcomas are a heterogeneous population of mesenchymal tumors that accounts for approximately 1% of all malignancies (1). Among them, fibrosarcoma originates from transformed spindle shaped fibroblasts, it occurs as a soft-tissue mass or as a primary or secondary bone tumor, and is predominantly located in deep soft tissue or adjacent to bones. Depending on the clinical presentation, fibrosarcomas can be divided into infantile/congenital fibrosarcoma, a low malignant/rarely metastasizing tumor, and adult-type fibrosarcoma, which occurs mainly between 30 and 60 years of age with more aggressive and malignant features (2).

As with all soft-tissue and bone sarcomas, the mainstay of fibrosarcoma treatment consists in the complete excision with an adequate margin, with prognostic projection depending on size and location of the tumor, histologic grade, and the presence of metastatic disease. For instance, high-grade primary fibrosarcoma and secondary fibrosarcoma have a 10-year survival rate lower than 30 and 10%, respectively (3, 4). Fibroblast growth factor-2 (FGF2) is the prototypic member of the FGF gene family that exerts its activity by binding to tyrosine kinase FGF receptors (FGFRs) expressed by four distinct FGFR1-4 genes (5). FGF2 is produced by different cell types (including endothelial cells, immune cells, fibroblasts, and cancer cells) and plays pleiotropic roles in different settings, including inflammation, tissue remodeling, wound repair and cancer (6, 7). In general, FGF production/release promotes the activation of autocrine and paracrine loops of stimulation driving important cellular/tissue processes like differentiation and proliferation. In cancer, different tumor cell lines express different members of the FGF family, including FGF2, and/or display an aberrant activation of the FGF/FGFR system (6, 8).

Normal fibroblasts produce FGF2 and its overexpression triggers their transformation *in vitro* (9). Indeed, in a transgenic murine model of dermal fibrosarcoma, FGF2 is actively produced by fibroblasts at all stages along their malignant transformation (mild fibromatosis, aggressive fibromatosis, and fibrosarcoma) and its expression correlates with the angiogenic phenotype of the tumor (10). Moreover, the forced expression of FGF2/Platelet-derived growth factor-BB in fibrosarcoma cells increases their aggressiveness, thus promoting neovascularization and a higher metastatic potential (11). On these bases, given its role on fibroblast transformation and its impact on tumor vascularization, the FGF2/FGFR system might represent a promising target for fibrosarcoma therapy.

The soluble pattern recognition receptor long pentraxin-3 (PTX3) is a member of the pentraxin family produced locally in response to inflammatory signals by different cell types, including various myeloid cells, vascular and lymphatic endothelial cells, epithelial cells, and mesenchymal cells (including fibroblasts) (12). PTX3 has been shown to play non-redundant functions in various physiopathological conditions, including angiogenesis and cancer (13). A unique N-terminal domain determines specific functions of PTX3, including its FGF-binding and inhibitory capacity (14, 15), whereas its C-terminal domain contains the pentraxin signature shared with the other family members (16). The anti-angiogenic/anti-tumor potential of PTX3 has been demonstrated in different types of FGF-dependent tumors, including melanoma, prostate and lung cancer (17–20). Moreover, PTX3 has been proposed as an extrinsic oncosuppressor, able to affect tumor-promoting inflammation mediated by complement and macrophages (21). Indeed, genetic loss of PTX3 in *PTX3*^−/−^ mice increases the incidence of cancer development and growth in carcinogen-induced models of fibrosarcoma and skin cancer (21). In addition, when compared to their normal tissue counterpart, different types of mesenchymal and epithelial tumors are characterized by loss/reduced expression of PTX3 due to epigenetic regulation of the gene (21, 22). For instance, high methylation levels of the *PTX3* gene promoter occur in mesenchymal cancers, including angiosarcoma, synovial sarcoma, leiomyosarcoma and chordoma (21).

Here, we demonstrate that PTX3 overexpression significantly reduced the proliferative and tumorigenic potential of fibrosarcoma cells *in vitro* and *in vivo*. In addition, PTX3 overexpression driven by the Tie2 promoter inhibits the growth of syngeneic fibrosarcoma tumor grafts in transgenic mice. In a translational perspective, the PTX3-derived small molecule chemical FGF trap NSC12 prevented the activation of the FGF/FGFR system in fibrosarcoma cells and reduced their tumorigenic activity *in vivo*. In conclusion, impairment of the FGF/FGFR system by FGF trap molecules may represent a novel therapeutic approach for the treatment of fibrosarcoma.

## Materials and methods

### Cell culture

Human HT-1080 and murine MC-TGS17-51 (MC17-51) fibrosarcoma cells from American Type Culture Collection (ATCC) were cultured in DMEM (Gibco) containing penicillin/streptomycin (100 U and 10 mg/mL, respectively) and supplemented with 10% fetal bovine serum (FBS-Gibco) at 37°C with 5% CO_2_. Fibrosarcoma cells were transfected with a pBABE-Puro vector harboring the full length human PTX3 cDNA (GenBank accession n° X63613) or a pBABE-Puro empty vector (Mock) using FuGENE (Promega). HT-1080 and MC17-51 stable transfectants were selected in the presence of 1.0 or 4.0 μM puromycin, respectively.

### Western blot analysis

Fibrosarcoma cells harvested from 80 to 90% confluent monolayers were homogenized in RIPA buffer containing 1% Triton-X100, 0.2% BriJ, 1.0 mmol/L sodium orthovanadate and protease inhibitors cocktail (Sigma). Protein concentrations were determined using the Bradford protein assay (Bio-Rad Laboratories, Milano, Italy). The expression of PTX3 was detected using a rabbit polyclonal anti-PTX3 antibody from B. Bottazzi (Humanitas Clinical Institute, Milan, Italy).

The analysis of FGFR signaling was performed using anti-pFGFR1 and anti-pFGFR3 antibodies (Santa Cruz Biotechnology) and anti-pFRS2, anti-pAKT, anti-pERK_1/2_ antibodies (Cell Signaling), and normalized with an anti-α-GAPDH antibody (Santa Cruz Biotechnology).

### PCR analysis

The expression of *PTX3, FGF2*, and *FGFR1-4* was evaluated by RT-PCR. Total RNA was extracted from 80% confluent fibrosarcoma cell monolayers cultured in complete growth media using the TRIzol® reagent (Invitrogen), following manufacturer's recommendations. Two microgram of total RNA were retro-transcribed using ImProm-IITM reverse transcriptase kit (Invitrogen) and oligo(dT)20 primers (IDT). The cDNA was used as template in PCR reactions using specific primers (see Supplementary Table 1).

### Cell proliferation

Cells were seeded (5 × 10^3^) in 48-well cell culture plates in complete medium. At 24, 48, and 72 h, cells were detached and absolute cell counts were obtained by using the MACSQuant Analyzer (Miltenyi Biotec) and normalized in respect to time 0. Human HT-1080 fibrosarcoma cells were seeded (5 × 10^3^) in 48-well cell culture plates in complete medium, starved in 1% FBS for 24 h, and treated with DMSO or different concentration of NSC12 (0,1- 1- 3- 6- 10- 20 μM). At 24 or 48 h cells were detached, counted after propidium iodide labeling using the MACSQuant Analyzer (Miltenyi Biotec) and normalized in respect to DMSO-treated cells.

### Clonogenic assay

Cells were seeded (3 × 10^2^) in 6-well cell culture plates and incubated in complete growth medium until visible colonies were formed (approximately 10 days). Then, the supernatant was removed and cells were stained for 20 min with a solution containing 0.1% crystal violet/20% methanol. After the removal of the staining solution, plates were photographed and colonies were counted by using the Image J software. Then, a 1% SDS solution was added to each well and the plates were incubated overnight at room temperature. Solution absorbance was measured at 595 nm using a spectrophotometer.

### Soft agar assay

Cells (5 × 10^4^) were suspended in 3 ml of complete growth medium containing 0.3% agar and poured onto 2 ml pre-solidified 0.6% agar in a 6-well plate. After 3 weeks of incubation, colonies were observed under a phase contrast microscope, photographed, and their area was measured using the ImageJ Software and the SA_NJ algorithm (23).

### *In vivo* studies

Animal experiments were approved by the local animal ethics committee (OPBA, Organismo Preposto al Benessere degli Animali, Università degli Studi di Brescia, Italy) and were performed in accordance with national guidelines and regulations. Procedures involving animals and their care conformed with institutional guidelines that comply with national and international laws and policies (EEC Council Directive 86/609, OJ L 358, 12 December 1987) and with “ARRIVE” guidelines (Animals in Research Reporting *in vivo* Experiments).

Seven-week-old NOD/Scid and C57BL/6 male mice were injected subcutaneously (s.c.) into the dorsolateral flank with mock and PTX3-transfected human HT-1080 (3 × 10^6^) and murine MC17-51 (1 × 10^6^) cells, respectively. In an additional experiment, wild-type and transgenic TgN(Tie2-hPTX3) mice (17) were injected s.c. with 10^6^ wild type MC17-51 cells.

For therapeutic treatment, 7-week-old NOD/Scid male mice were injected s.c. with HT-1080 cells (3 × 10^6^). When tumors were palpable (approximately 40 mm^3^), mice were treated intraperitoneally (i.p.) with vehicle (DMSO) or NSC12 (7.5 mg/kg) every other day. Tumors were measured with calipers and the volume was calculated according to the formula *V* = (D × d2)/2, where D and d are the major and minor perpendicular tumor diameters, respectively. Tumor volume data were analyzed with a 2-way analysis of variance, and individual group comparisons were evaluated by the Bonferroni correction. At the end of the experimental procedure, tumors were surgically removed, weighed and paraffin embedded for immunohistochemical analysis.

### Immunohistochemistry

Formalin-fixed, paraffin-embedded samples were sectioned at a thickness of 3 μm, dewaxed in xylene, hydrated and stained with hematoxylin and eosin (H&E) for histological analysis or alternatively processed for immunohistochemistry.

The following primary antibodies were used: rabbit polyclonal anti-human PTX3 (kind gift of B. Bottazzi, Humanitas Clinical Institute-Milan), rat monoclonal anti-mouse CD31 (Dianova), rat monoclonal anti-mouse Ki67 (Dako), rabbit anti-phospho-Histone H3 (Ser10) (Millipore). Sections were then incubated with HRP labeled polymer anti-rabbit or with biotinylated anti-rat secondary antibody and subsequently in Vectastain Elite ABC kit (Vector Laboratories). Positive signal was revealed by 3,3′-diaminobenzidine staining (Roche) and counterstained with Carazzi's haematoxylin to identify nuclei, dehydrated and mounted in DPX (Sigma) before analysis by light microscopy. Images were acquired with the automatic high-resolution scanner Aperio System (Leica Biosystems, Wetzlar, Germany, EU).

## Results

### PTX3 impairs the growth of fibrosarcoma cells *in vitro*

The FGF/FGFR system plays a crucial role in fibroblast transformation and fibrosarcoma onset and progression (9, 10). Accordingly, human HT-1080 and murine MC17-51 fibrosarcoma cells express FGF2 and different FGFRs (Supplementary Figure 1).

PTX3 has been described as a potent FGF2 inhibitor (14, 18, 19) and has been reported to be down-modulated in different types of cancer, including soft tissue sarcomas (21, 22) in which its absence exerts pro-tumor effects (13, 21). In line with literature data (21), HT-1080 and MC17-51 cells express negligible levels of PTX3 due to an epigenetic regulation of the gene whose expression can be upregulated following treatment with demethylating agents (Supplementary Figure 2).

**Figure 1:**
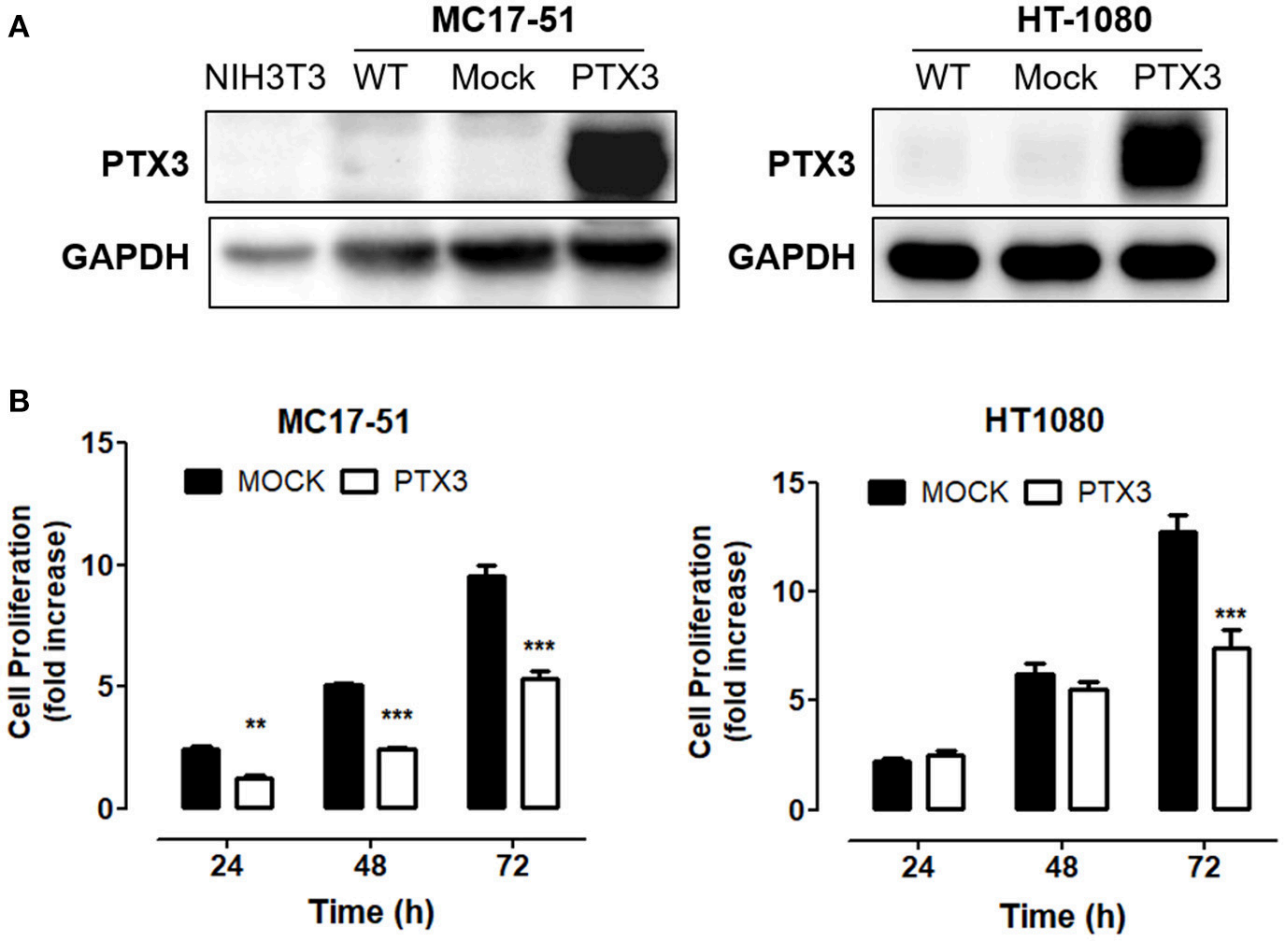
PTX3 impairs fibrosarcoma proliferation *in vitro*. **(A)** Western blot analysis of PTX3 expression in wild type (WT), empty vector- transduced (Mock), and PTX3-overexpressing (PTX3) MC17-51 and HT-1080 cells. **(B)** Proliferation of control (mock) and PTX3-overexpressing (PTX3) MC17-51 and HT-1080 cells at 24, 48, and 72 h. Cell proliferation is expressed as fold increase in respect to the number of seeded cells; data are the mean ± S.E.M of 3 independent determinations (^**^P < 0.01, ^***^P < 0.001, Student's *t*-test).

On this basis, murine MC17-51 and human HT-1080 cells were transfected with a pBABE-Puro vector harboring the full-length human PTX3 cDNA whereas an empty vector was used to generate control (mock) cell populations. As a result, wild type and mock MC17-51 and HT-1080 cell populations express very low levels of PTX3 protein, whereas stable PTX3 transfectants produce and released significant amounts of PTX3 (Figure 1A and Supplementary Figure 3).

**Figure 2:**
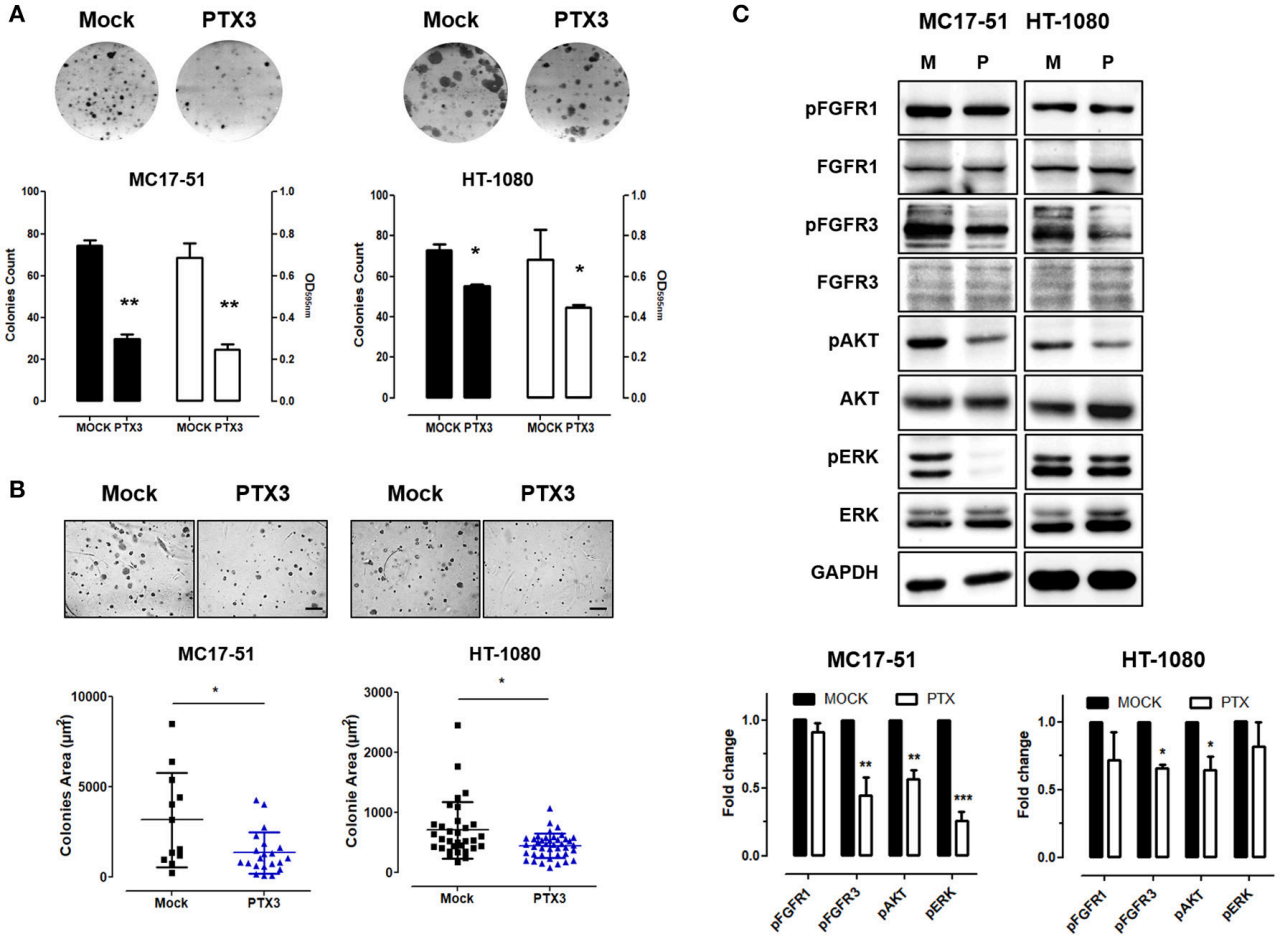
PTX3 overexpression reduces the clonogenic capacity, anchorage independent growth and FGF/FGFR signaling in fibrosarcoma cells. **(A)** Mock- and PTX3-transfected MC17-51 and HT-1080 cells were seeded at low density. After 10 days, cell colonies were stained with crystal violet (upper representative images) and counted (black bars) or solubilized in 1% SDS and solution absorbance was measured at 595 nm using a spectrophotometer (white bars). **(B)** Transfectants were grown in soft agar for about 3 weeks, cell colonies were photographed (upper representative images) and their area (in μm^2^) was measured. Scale bar: 0.25 mm. **(C)** Western blot and corresponding densitometric analysis of FGF/FGFR signaling in mock- and PTX3- transduced MC17-51 and HT-1080 cells. *In vitro* data are the mean ± S.E.M of triplicate observations; Western blot analysis was performed twice; ^*^P < 0.05; ^**^P < 0.01, ^***^P < 0.001 Student's *t*-test.

As shown in Figure 1B, PTX3 overexpression significantly reduced the proliferation rate of both murine and human fibrosarcoma transfectants when compared to controls, with a significant decrease of their clonogenic capacity (Figure 2A). In addition, PTX3 overexpression reduced the anchorage-independent ability of both murine and human transfectants to form 3D colonies in soft agar (Figure 2B).

In keeping with its FGF trapping antagonist potential (13, 14, 17), the inhibitory activity exerted by PTX3 overexpression in fibrosarcoma cells is paralleled by a significant decrease of FGFR3 phosphorylation, as well as of the activation of mitogen activated protein kinase Erk_1/2_ and AKT in murine MC17-51 cells, and of FGFR3/Akt activation in HT-1080 cells (Figure 2C).

At variance, no significant differences were observed between mock- and PTX3-trasfected MC17-51 and HT-1080 cells for their capacity to repair a wounded cell monolayer (Supplementary Figure 4), thus indicating that PTX3 overexpression does not affect the motility of fibrosarcoma cells whose migratory capacity might be regulated by FGF/FGFR independent signaling pathways.

### PTX3 impairs fibrosarcoma growth *in vivo*

**Figure 3:**
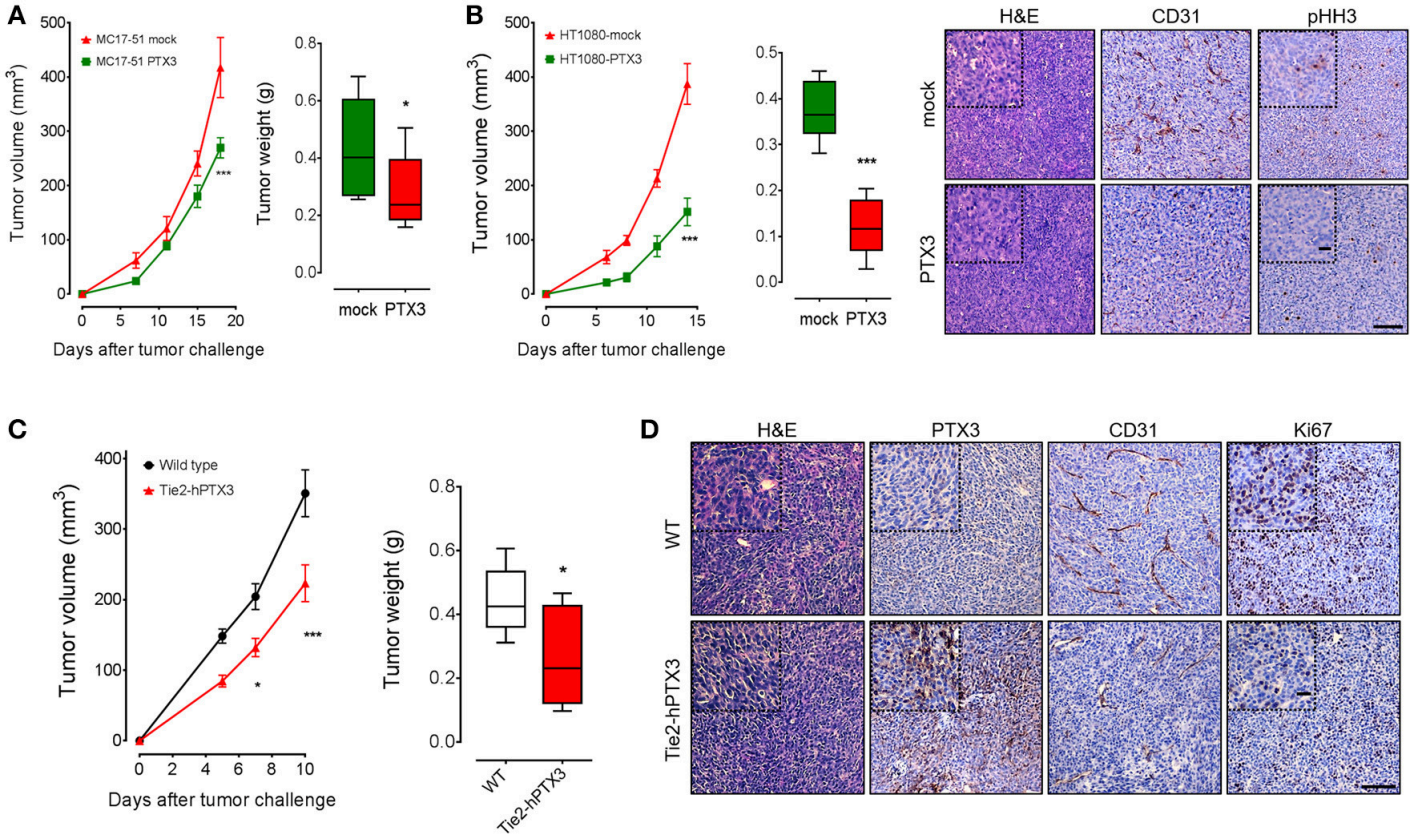
PTX3 impairs the growth of fibrosarcoma cells *in vivo*. Mock and PTX3-transfected MC17-51 and HT-1080 cells were injected s.c. in syngeneic C57BL/6 **(A)** or immune-compromised NOD/Scid **(B)** mice (*n* = 10 mice/group), respectively. Tumors were measured with caliper at different time points and weighted at the end of the experiment. HT-1080 tumors were stained for phospho-Histone H3 (pHH3) and CD31 (main scale bar, 200 μm; magnification insert: scale, bar 50 μm). **(C)** Wild type MC17-51 cells were injected s.c. in wild type (C57BL/6) and transgenic (Tie2-hPTX3) mice (*n* = 10 mice/group). **(D)** Explanted tumors were immunostained with anti-PTX3, anti-CD31 and anti-Ki67 antibodies (main scale bar, 200 μm; magnification insert: scale bar 50 μm). Data are the mean ± S.E.M of 10 tumors per group. (^*^P < 0.05; ^***^P < 0.001).

To assess the effect of PTX3 on the tumorigenic activity exerted by fibrosarcoma cells *in vivo*, mock- and PTX3-overexpressing MC17-51 and HT-1080 cells were injected s.c. in the flank of C57BL/6 and NOD/Scid mice, respectively. As shown in Figures 3A,B, PTX3 overexpression caused a significant delay of tumor growth in both murine and human tumor grafts. Accordingly, the average weight of harvested PTX3-overexpressing MC17-51 and HT-1080 tumors was significantly reduced when compared to mock derived lesions (Figures 3A,B) and was also accompanied by a reduction in pHH3^+^ proliferating cells and of CD31^+^ tumor vessels (Figure 3B).

On this basis, to assess the effect of the local/systemic delivery of PTX3 protein on fibrosarcoma growth, wild type MC17-51 cells were injected in syngeneic wild type (C57BL/6) and transgenic TgN(Tie2-hPTX3) mice that overexpress human PTX3 under the endothelial specific Tie2 promoter (17). In line with the *in vivo* data herewith obtained with fibrosarcoma PTX3 transfectants (see above) and previous observations on different FGF-dependent tumor types grafted in TgN(Tie2-hPTX3) mice (17, 24), endothelial expression and stroma accumulation of PTX3 significantly impaired the growth of fibrosarcoma MC17-51 tumor grafts in these animals. This resulted in reduced average weight of the lesions harvested from transgenic mice when compared to wild type animals (Figure 3C). Again, immunohistochemical analyses performed on tumor samples revealed that tumors grafted in TgN(Tie2-hPTX3) mice showed a decrease of cell proliferation (expressed as Ki67^+^ areas) and of vascularization (CD31^+^ areas) when compared to control animals (Figure 3D). Thus, similar to the results obtained following PTX3 expression in tumor cells, the systemic production of PTX3 in transgenic TgN(Tie2-hPTX3) mice results in a significant inhibition of both tumor cells as well as of different FGF/FGFR-dependent components of tumor microenvironment.

### The PTX3-derived small molecule FGF trap impairs fibrosarcoma growth *in vitro* and *in vivo*

**Figure 4:**
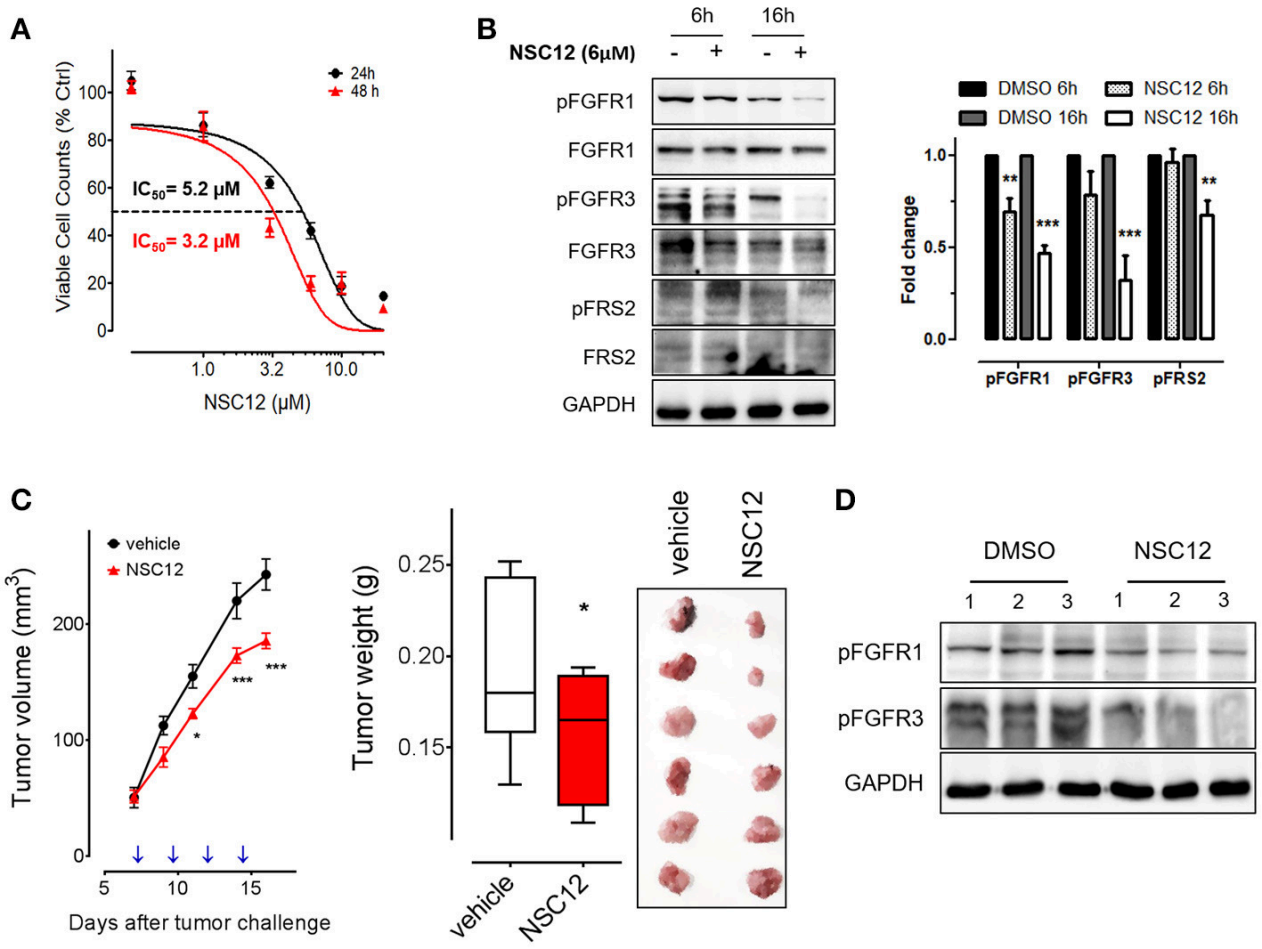
The FGF trap small molecule SNC12 decreases cell viability, FGF/FGFR signaling and tumorigenic activity of human fibrosarcoma cells.**(A)** Proliferation of HT-1080 cells treated for 24 or 48 h with increasing concentrations of NSC12. Data are expressed as percentage of viable cells in respect to DMSO-treated control cells. **(B)** Western blot and densitometric analysis of FGF/FGFR signaling in HT-1080 cells after 6 or 16 h treatment with 10 μM NSC12; Western blot analysis was performed twice; ^**^P < 0.01; ^***^P < 0.001, Student's *t*-test. **(C)** HT-1080 cells were injected s.c in NOD/Scid mice and animals were treated with 7.5 mg/kg of NSC12 or with DMSO (*n* = 10 mice/group) at the indicated time points (arrows). Tumors were measured with caliper (left panel) and weighted at the end of the experiment (middle panel). Representative tumors are shown in the right panel. Data are the mean ± S.E.M of 10 tumors per group. (^*^P < 0.05; ^***^P < 0.001). **(D)** Western blot analysis of representative HT-1080 tumors treated with DMSO (vehicle) or NSC12.

The PTX3-derived small molecule NSC12 represents the first low molecular weight FGF-trap endowed with significant implications for the therapy of FGF-dependent tumors (17, 24). Thus, in a translational effort to exploit the FGF-blocking activity exerted by PTX3 on fibrosarcoma cells, we evaluated the effect of this novel FGF trap on the tumorigenic potential of human HT-1080 cells *in vitro* and *in vivo*. As shown in Figure 4A, NSC12 inhibits the proliferation of HT-1080 cells with an IC_50_ equal to approximately 5.2 μM and 3.2 μM after 24 and 48 h of treatment, respectively. Accordingly, the activation of FGFR1, FGFR3 and of the Fibroblast Growth Factor Receptor Substrate 2 (FRS2) is significantly reduced following 6 and 16 h of treatment with NSC12 (Figure 4B). On this basis, HT-1080 cells were injected in immunocompromised mice and, once tumors were palpable, animals were treated i.p. every other day with vehicle or 7.5 mg/kg of NSC12 (17). As shown in Figure 4C, treatment with NSC12 significantly affects the rate of growth of HT-1080 tumors and the average weight of harvested lesions when compared to vehicle. Accordingly, Western blot analysis on representative explanted tumors confirmed a significant reduction of FGFR1 and FGFR3 activation following treatment with NSC12 (Figure 4D).

## Discussion

Fibrosarcoma is a rare and highly malignant mesenchymal tumor originating from transformed/hyper-proliferating spindle-shaped fibroblasts (25). Since other spindle-cell shaped sarcomas exist, its identification is based on a diagnosis of exclusion, and the WHO classification of soft tissue sarcomas includes fibrosarcoma as part of the fibroblastic/myofibroblastic sarcomas (26). In general, fibrosarcoma can be divided in infantile/congenital type fibrosarcoma, with intermediate malignant and rarely metastasizing features, and adult-type fibrosarcoma, a highly malignant tumor.

Even though fibrosarcomas occurring in adults represent only 3.6% of all adult sarcomas (27), the therapeutic options are limited and prognosis is generally poor. Indeed, despite the histopathological grading is considered the most important prognostic indicator, regardless of grade, the 5-year survival rate is around 40–60% and the 10- year survival rate is 60% for low-grade, and 30% for high-grade tumors (28, 29).

To date, surgery represents the standard therapy of localized soft tissue sarcomas together with radiotherapy and chemotherapy, but a high number of fibrosarcoma patients are low or non-responder to radiotherapy and develop multidrug resistance to chemotherapy (30).

On these bases, tumor heterogeneity and microenvironment are under investigation in soft tissue sarcomas, and in particular in fibrosarcoma, to investigate new therapeutic perspectives that take into consideration tumor stemness, malignant fibroblast differentiation and extracellular matrix/stromal components.

The FGF/FGFR system is a key player in the tumor/microenvironment interplay (5, 31) and its aberrant activation consequent to FGF2 overexpression may trigger fibrosarcoma progression by facilitating fibroblast transformation and increasing fibrosarcoma aggressiveness, neovascularization and metastatic potential (9, 11). In addition, *FGFR1* has been shown to represent a driver gene in multiple soft tissue sarcoma subtypes characterized by its amplification/overexpression, thus representing a potential therapeutic target in these tumors (32). Notably, the multi-target tyrosine kinase inhibitor AL3818 (anlotinib), targeting also FGFR1/2/3 (33), is in phase III trial for metastatic/advanced alveolar soft part sarcoma, leiomyosarcoma and synovial sarcoma (NCT03016819).

Here, we demonstrate that FGF trap molecules can hijack the FGF/FGFR system to reduce the tumorigenic potential of murine and human fibrosarcoma cells *in vitro* and *in vivo*. The pattern recognition receptor PTX3 has been proposed as an extrinsic oncosuppressor, genetic loss of PTX3 in *Ptx3*^−/−^ mice increasing tumor growth in carcinogen-induced models of fibrosarcoma and skin cancer (21). Accordingly, homozygous PTX3 inactivation in *Ptx3*^−/−^ mice enhances FGF-dependent angiogenesis, tumor growth and metastasis (17). When assessed for the capacity to interact with a variety of extracellular signaling polypeptides, PTX3 was found to bind FGF2, thus inhibiting FGF2-dependent endothelial cell proliferation *in vitro* and angiogenesis *in vivo* (14, 15, 34, 35). In addition, PTX3 binds other members of the FGF family *via* its N-terminal extension, including FGF6, FGF8b, FGF10, and FGF17 (19). Accordingly, transgenic PTX3 overexpression by tumor cells efficaciously impairs the activation of the FGF/FGFR system in FGF-driven tumor cell lines, thus affecting tumor growth and metastasis in different models of melanoma, prostate and mammary carcinomas (18–20). Moreover, PTX3 accumulation in tumor stroma and bloodstream, obtained through endothelial specific overexpression of PTX3 in transgenic mice, deeply affects the tumorigenic, angiogenic and metastatic potential of various syngeneic FGF-dependent tumor cell lines (17). Our study shows for the first time that PTX3 overexpression hampers the tumorigenic potential of fibrosarcoma cells and that stromal PTX3 accumulation in transgenic TgN(Tie2-hPTX3) mice significantly inhibits the growth of syngeneic fibrosarcoma tumor grafts.

Despite its oncosuppressive activity, the complex proteinaceous structure of PTX3 limits its clinical/pharmacological exploitation. In order to overcome these limitations, NMR data about FGF2/PTX3 interaction and pharmacophore modeling of a minimal PTX3-derived FGF2-binding pentapeptide were used for the identification of NSC12 as the first small molecule able to act as an extracellular pan-FGF trap and endowed with a significant activity against various FGF-dependent tumor types (17, 36).

In keeping with the inhibitory activity exerted by PTX3 on fibrosarcoma cells, and with the relevance of the FGF/FGFR system in fibrosarcoma biology, treatment with the PTX3-derived FGF-trap NSC12 causes a significant inhibition of the tumorigenic potential of HT-1080 cells *in vitro* and *in vivo*, paralleled by a decrease of FGFR activation and signaling in these cells.

Together, our data provide novel evidence about the non-redundant role of the FGF/FGFR system in fibrosarcoma and point to “FGF trapping” as a novel strategy for the therapy of this neoplasia. In this frame, the PTX3-derived small molecule FGF-trap NSC12 may pave the way to the discovery of novel drug candidates for those subsets of soft tissue sarcomas, including fibrosarcomas, in which FGFR ligands play an onco-driving role.

## Author contributions

PR, SM, and JS performed *in vitro* experiments. FM performed *in vivo* experiments. EF and AG performed the IHC experiments, MP revised the paper. AD conceived the experiments and revised the paper. RR conceived and supervised the experiments, and wrote the paper.

### Conflict of interest statement

The authors declare that the research was conducted in the absence of any commercial or financial relationships that could be construed as a potential conflict of interest.
