# Supplementary material for: Long Pentraxin 3-Mediated Fibroblast Growth Factor Trapping Impairs Fibrosarcoma Growth
Source: Front Oncol. 2018 Nov 1;8:472. doi: 10.3389/fonc.2018.00472 (PMC6221954; doi:10.3389/fonc.2018.00472)
Supplement: Supplementary file 1 [file Data_Sheet_1.PDF]

Supplementary Material

Long Pentraxin 3-mediated Fibroblast Growth Factor trapping impairs fibrosarcoma growth

Priscila Fabiana Rodrigues, Sara Matarazzo, Federica Maccarinelli, Eleonora Fogli2, Arianna Giacomini, João Paulo Silva Nunes, Marco Presta, Adriana Abalen Martins Dias, Roberto Ronca

Correspondence:

Ronca Roberto: roberto.ronca@unibs.it

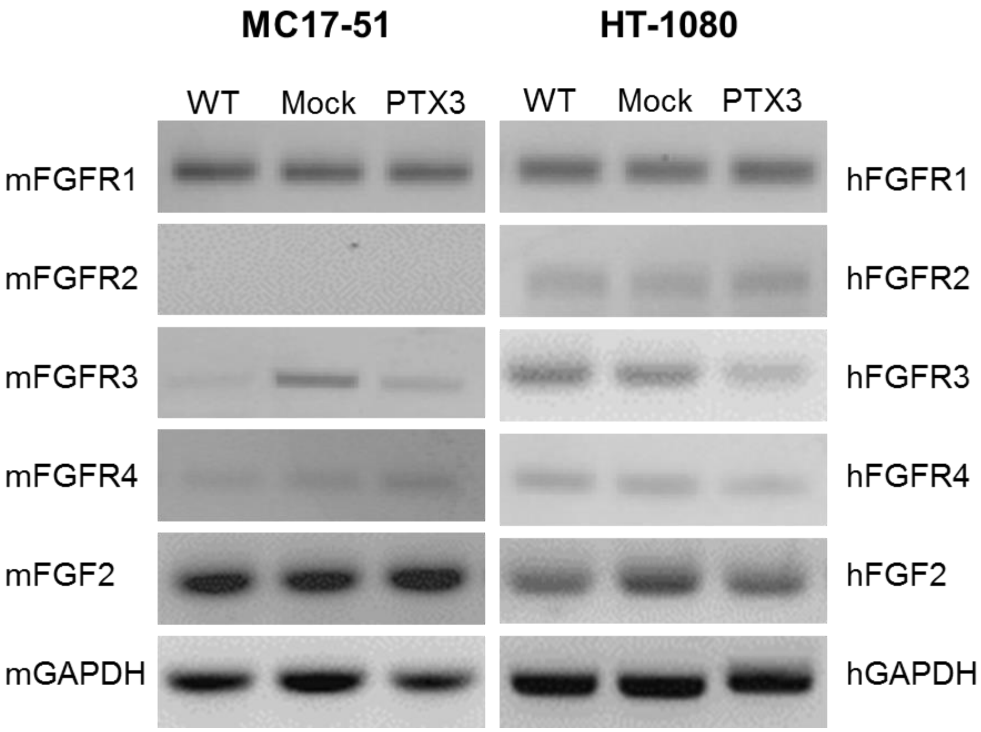

Supplementary Figure 1. Expression of the FGF/FGFR system in MC17-51 and HT1080 cells and transfectants. Semi-quantitative PCR analysis of the expression of murine (m) or human (h) FGFR1/2/3/4 and FGF2 in MC17-51 and HT-1080 cells wild type (WT) transfected with empty vector (Mock) or overexpressing PTX3 (PTX3). PCR was performed using specific primers indicated in Supplementary Table 1, up to 35 cycles of amplifications.

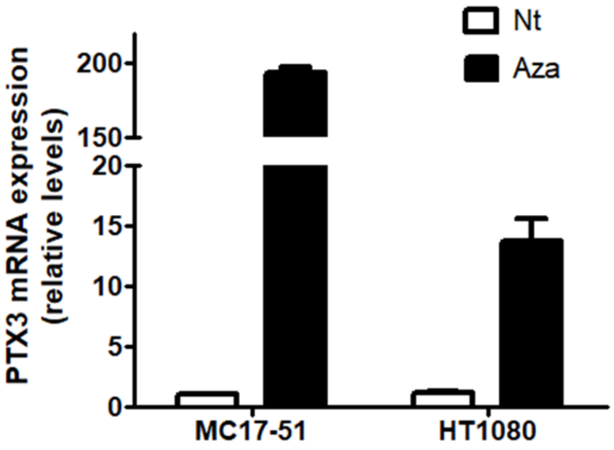

Supplementary Figure 2. PTX3 expression is silenced by methylation in fibrosarcoma cells. MC17-51 and HT-1080 cells were treated with the demethylating agent 5-aza-2'-deoxycytidine (Aza) at the concentration of 1 µM and 6 µM, respectively. Total RNA was extracted, retro-transcribed and cDNA used as template in quantitative PCR reaction to measure PTX3 expression levels in respect to untreated cells (Nt).

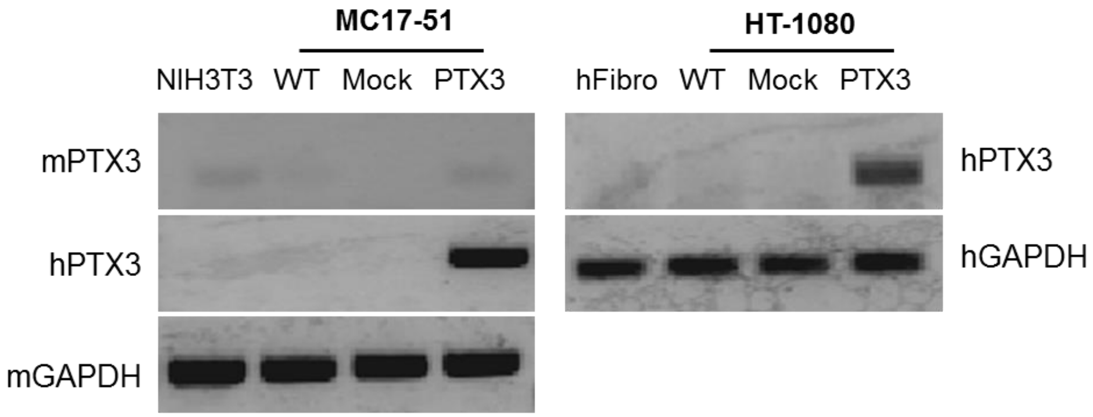

Supplementary Figure 3. Expression of the PTX3 in fibroblasts and in MC17-51 and HT1080 cells and transfectants. Semi-quantitative PCR analysis of the expression of murine (m) or human (h) PTX3 in murine (NIH3T3) and human (hFibro) fibroblasts, and in fibrosarcoma (MC17-51 and HT-1080) cells wild type (WT), transfected with empty vector (Mock) or overexpressing PTX3 (PTX3). PCR was performed using specific primers indicated in Supplementary Table 1, up to 35 cycles of amplifications.

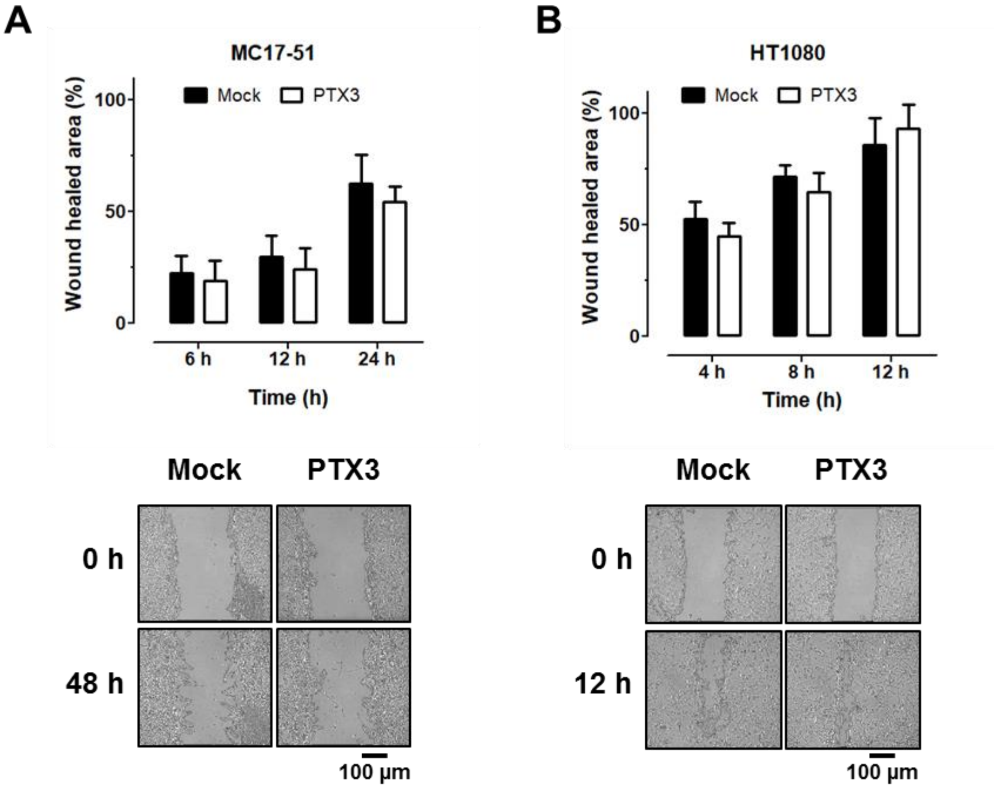

Supplementary Figure 4. PTX3 overexpression does not affect the motility of fibrosarcoma cells. Confluent monolayers of mock and PTX3-transfected MC17-51 (A) and HT-1080 (B) cells were seeded in 24 well plates in medium without fetal bovine serum and were scraped with a 200 µl tip to obtain two 2 mm thick denuded areas in each well. At different time points, wounds were photographed and the percentage of healed area was measured with the ImageJ support and WH_NJ algorithm [1]. Black bars: mock cells; white bars: PTX3-overexpressing cells. Representative images of the corresponding wounds are shown in the lower panels

1. Nunes, J.P.S. and A.A.M. Dias, ImageJ macros for the user-friendly analysis of soft-agar and wound-healing assays. Biotechniques, 2017. 62(4): p. 175-179.

Supplementary Table 1. Primer sequences.

	Primer forward	Primer reverse
**mouse PTX3**	GACCTCGGATGACTACGAG	CTCCGAGTGCTCCTGGCG
**human PTX3**	GGTCTGCAGTGTTGGCCGAGAA	TCGTCCGTGGCTTGCAGCAG
**mouse FGF2**	TTCAAGGACCCCAAGCGG	TGCCCAGTTCGTTTCAGTG
**human FGF2**	TCAAACTACAACTCCAAGCAGAA	GTAACACACTTAGAAGCCAGCA
**mouse FGFR1**	CAGATAACACCAAACCAAACCG	GACCGCTCCACGACATC
**human FGFR1**	GGAGGCTACAAGGTTCGCTAT	CATACGGCAAGTTGTCTGGC
**mouse FGFR2**	ACAAAGACAAGCCCAAGGAG	CGAAGGACCAGACATCACTC
**human FGFR2**	TCCATGAACTCCAACACCCC	TAGAGAGGTCCATCCTGCGT
**mouse FGFR3**	CAGGAGCAGTTGGTCTTCG	CCGCAGCTTGATGCCT
**human FGFR3**	CACAAGGTCTCTCGCTTCC	AGTCGCATCATCTTTCAGCAT
**mouse FGFR4**	GCTGTGAGAAGGAGATGCG	GTGTCCAGTAGGGTGCTTG
**human FGFR4**	GGGGTGTATCATCGGCAAGT	GTCGGAGGCATTGTCTTTCAG
**mouse GAPDH**	CTCTCTGCTCCTCCTGTT	GATGATGACCCTTTTGGCTC
**human GAPDH**	TTGTGGAAGGGCTCATGACCA	CGTATTCATTGTCATACCAGG
